# Functional heterogeneity in tumor-derived human pancreatic stellate cells: Differential expression of HGF and implications for mitogenic signaling and migration in pancreatic cancer cells

**DOI:** 10.18632/oncotarget.17800

**Published:** 2017-05-11

**Authors:** Vegard Tjomsland, Monica Aasrum, Thoralf Christoffersen, Ivar P Gladhaug

**Affiliations:** ^1^ Department of Pharmacology, Institute of Clinical Medicine, University of Oslo, Oslo, Norway; ^2^ Department of Hepato-Pancreato-Biliary Surgery, Institute of Clinical Medicine, University of Oslo, Oslo, Norway; ^3^ Department of Hepato-Pancreato-Biliary Surgery, Oslo University Hospital Rikshospitalet, Oslo, Norway

**Keywords:** pancreatic cancer, human pancreatic stellate cells, tumor-stroma interactions, HGF, cancer cell migration

## Abstract

The pancreatic stellate cell (PSC) is the principal cell type of the desmoplastic stroma of pancreatic ductal adenocarcinoma (PDAC). PSCs interact with cancer cells and influence the progression of the disease through a complex network of signaling molecules including hepatocyte growth factor (HGF). Functional heterogeneity of PSCs within a tumor might conceivably influence tumor progression. We investigated PSC populations isolated from different human PDACs and examined the effects of PSC-conditioned medium on BxPC-3 and AsPC-1 pancreatic cancer cells. The different PSC populations exhibited a wide range of variation (120−3,000 pg/ml) in their ability to secrete HGF. Media from high-HGF-producing PSCs stimulated phosphorylation of Met, Gab1, and ERK in the cancer cells and induced increases in DNA synthesis and migration which were blocked by the Met inhibitor SU11274, indicating a role of HGF as a mediator. HGF levels produced by PSCs and the effects of PSC media on the cancer cells were increased by IL-1α and inhibited by TGFβ. The functional heterogeneity of PSCs in terms of HGF-mediated tumor-stroma interactions suggests that inhibition of the HGF pathway as a novel treatment approach in PDAC might have different effects in different subsets of patients.

## INTRODUCTION

Pancreatic ductal adenocarcinoma (PDAC), generally referred to as pancreatic cancer, is a highly malignant disease associated with late diagnosis, early metastasis, limited response to chemotherapy, and an extremely poor prognosis [1]. Massive research has provided extensive knowledge about the molecular and cellular pathobiology of PDAC [2] but this has not yet been translated into significant therapeutic progress with improved clinical outcome. The 5-year survival rate is typically reported as less than 4% [3] and even for the 15–20% of patients for whom surgical resection can be offered, most patients develop disease recurrence within a year [4].

A characteristic feature of PDAC is a prominent desmoplastic stroma, a dense fibro-inflammatory microenvironment which surrounds islands of cancer cells and constitutes the bulk of the tumor mass [2]. It is composed of extracellular matrix (ECM) components and several stromal cells, of which the most abundant are the pancreatic stellate cells (PSCs) [5], a special type of cancer-associated fibroblasts. The PSCs are the major drivers and organizers of the desmoplastic reaction [6]. In the healthy pancreas they exist in a quiescent state but in response to various pathophysiological stimuli, notably the influence from emerging neoplastic ductal epithelium, the PSCs are activated, leading to myofibroblast-type cells with capacity to proliferate and secrete ECM proteins, such as collagen, fibronectin, and matrix metalloproteinases, as well as various growth factors and other active molecules [7]. While much evidence suggests that the desmoplastic stroma promotes and sustains pancreatic cancer [8], other findings have challenged this view and indicated that depletion of stroma with PSCs accelerate the progression of pancreatic carcinomas [9, 10]. With these discrepant data it is of importance to study further the interactions between the PSCs and the pancreatic carcinoma cells.

Hepatocyte growth factor (HGF) is a glycoprotein mainly expressed by mesenchymal cells, whereas Met, the receptor of HGF, is mainly expressed by epithelial cells [11]. Met has been found to be overexpressed in many human pancreatic cancer cell lines [12, 13]. Activation of Met by HGF has been shown to promote both growth and invasion of human pancreatic cancer cells [14]. Fibroblasts and pancreatic cancer cells can communicate through the HGF/Met pathway, as shown where HGF secreted by tumor-derived fibroblasts stimulated pancreatic cancer cell invasion [13]. Recent reports have also supported the ability of PSCs to secrete HGF, which in turn can stimulate proliferation and migration of vascular endothelial cells and pancreatic cancer cells [15, 16]. It has also been shown that HGF-secretion from fibroblasts is increased by IL-1α or by co-culture with IL-1α-expressing pancreatic cancer cells [17].

Heterogeneity among PSCs within the same tumor has been observed, suggesting that various subsets of PSCs have different effects on cancer cell migration and proliferation [18] and on outcome in patients [19]. Although relatively few data exist on functional heterogeneity of stromal cells, the possibility of heterogeneity among PSC populations isolated from different patients has been put forward, indicating that different PCS phenotypes could interact differently with the cancer cells in the tumor microenvironment [5]. In the present study, we aimed to determine the ability of PSC populations derived from different patients to stimulate DNA synthesis and migration in pancreatic cancer cells, and, specifically, to what extent HGF secretion from PSCs is involved in the effects. In addition, since TGFβ has an inhibitory role in the regulation of IL-1α-mediated cross-talk between pancreatic cancer cells and PSCs [20] we also examined how IL-1α and TGFβ can modulate the level of HGF expressed by the PSCs and how this affects cancer cell DNA synthesis and migration.

## RESULTS

### Characterization of pancreatic stellate cell phenotype

**Figure 1:**
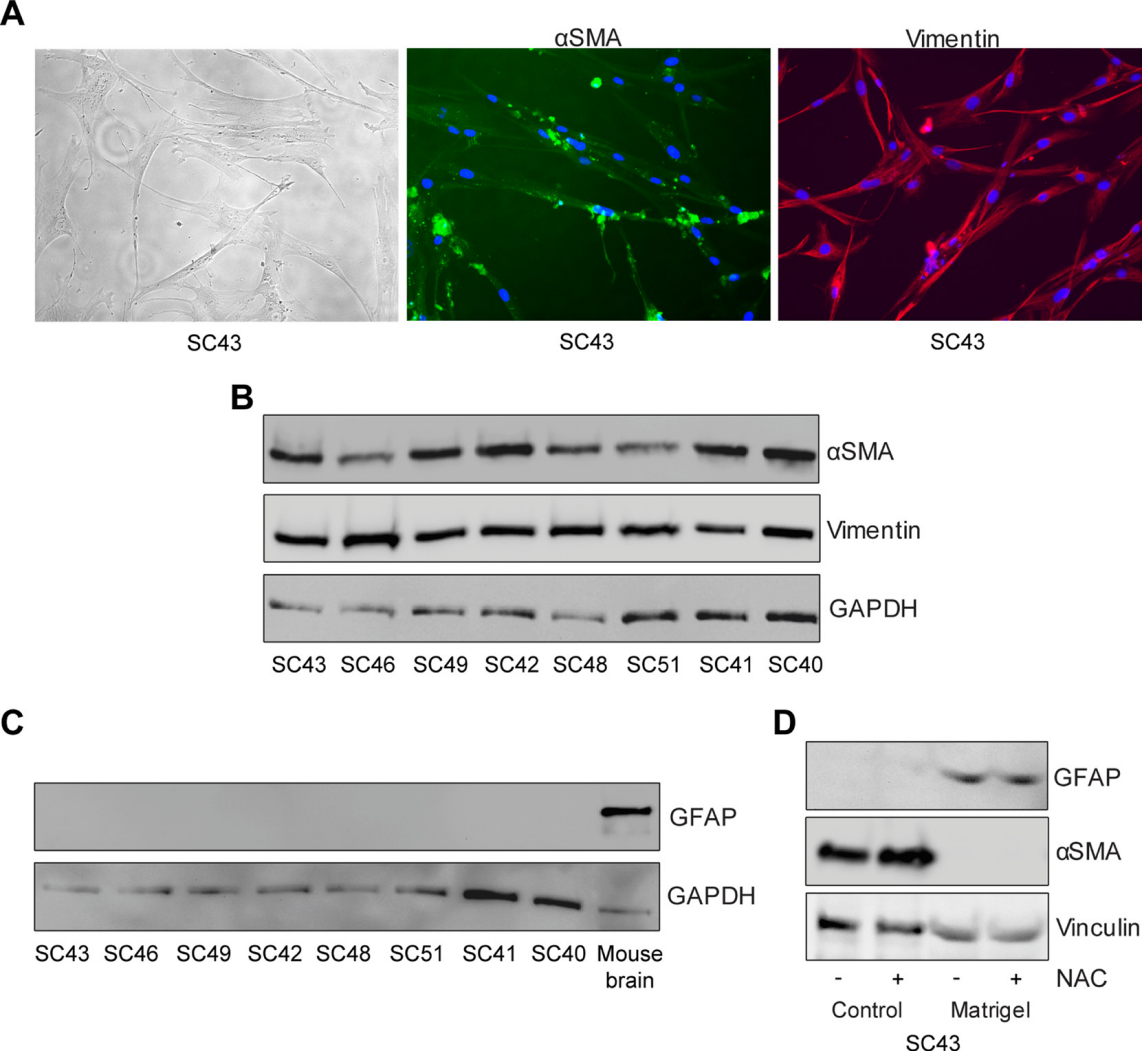
Phenotypic characterization of human pancreatic stellate cells (**A**) Pancreatic stellate cells were immunostained with anti-αSMA (green) and anti-Vimentin (red) antibodies. The stellate cells were lysed and proteins subjected to immunoblotting using (**B**) anti-αSMA, anti-Vimentin and (**C**) anti-GFAP antibodies. Mouse brain was used as a positive control for GFAP and anti-GAPDH was used as a loading control. (**D**) De-activation of pancreatic stellate cells was conducted by culturing the cells for six days in normal tissue culturing plates or on matrigel, with or without the addition of N-acetylcysteine (NAC). Thereafter, lysates from the cells were immunostained with anti-GFAP, anti-αSMA and anti-Vinculin (loading control) antibodies.

Primary cultures of PSCs were generated from surgical specimens from eight patients with pancreatic ductal adenocarcinoma. We performed immunofluorescence staining and found that the cells exhibited morphologies typical of pancreatic stellate cells with a fibroblast-like form and stained positive for αSMA and vimentin consistent with activated PSCs (Figure 1A). Western blotting confirmed expression of αSMA and vimentin in PSCs from all eight tumors (Figure 1B). No expression of GFAP was detected (Figure 1C), consistent with the reported loss of its expression during culturing of stellate cells [21]. Expression of GFAP was however re-established and the expression of αSMA was abolished when culturing the PSCs on matrigel (Figure 1D), confirming the phenotype of these cells as reported by others [22].

### Conditioned medium from PSCs stimulates DNA synthesis and migration of pancreatic cancer cells

**Figure 2:**
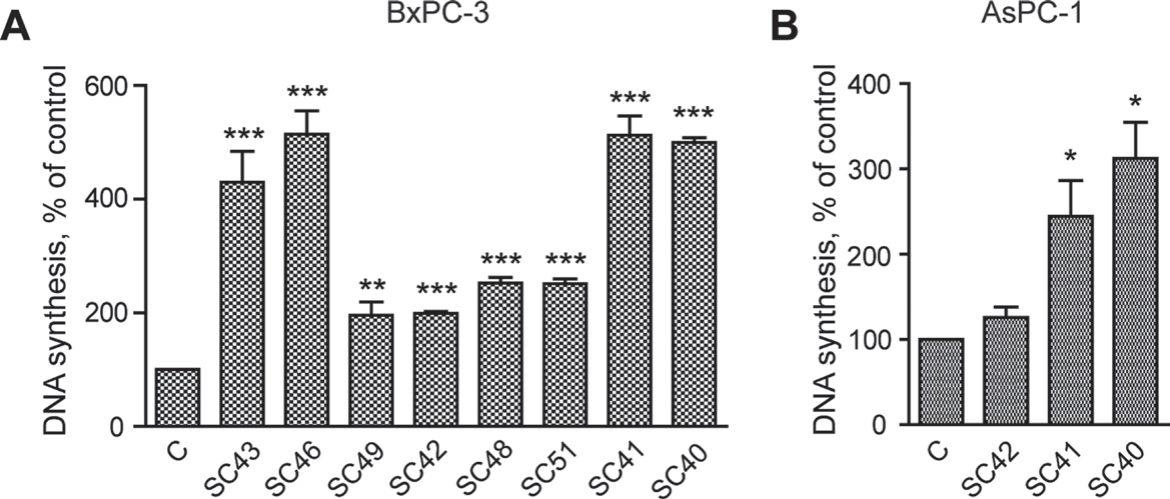
Conditioned medium from pancreatic stellate cells stimulate cancer cell DNA synthesis Effect of pancreatic stellate cells on pancreatic cancer cell DNA synthesis were utilized by measuring the [^3^H]-thymidine incorporation in BxPC-3 (**A**) and AsPC-1 (**B**) cells. The cancer cells were stimulated for 24 hours in PSC conditioned medium, with [^3^H]-thymidine added at 18 hours. [^3^H]-thymidine incorporation was measured and the results presented as mean +/−SEM normalized to unstimulated control. Error bars represent S.E.M.; *p < 0.05, **p < 0.005, ***p < 0.001.

**Figure 3:**
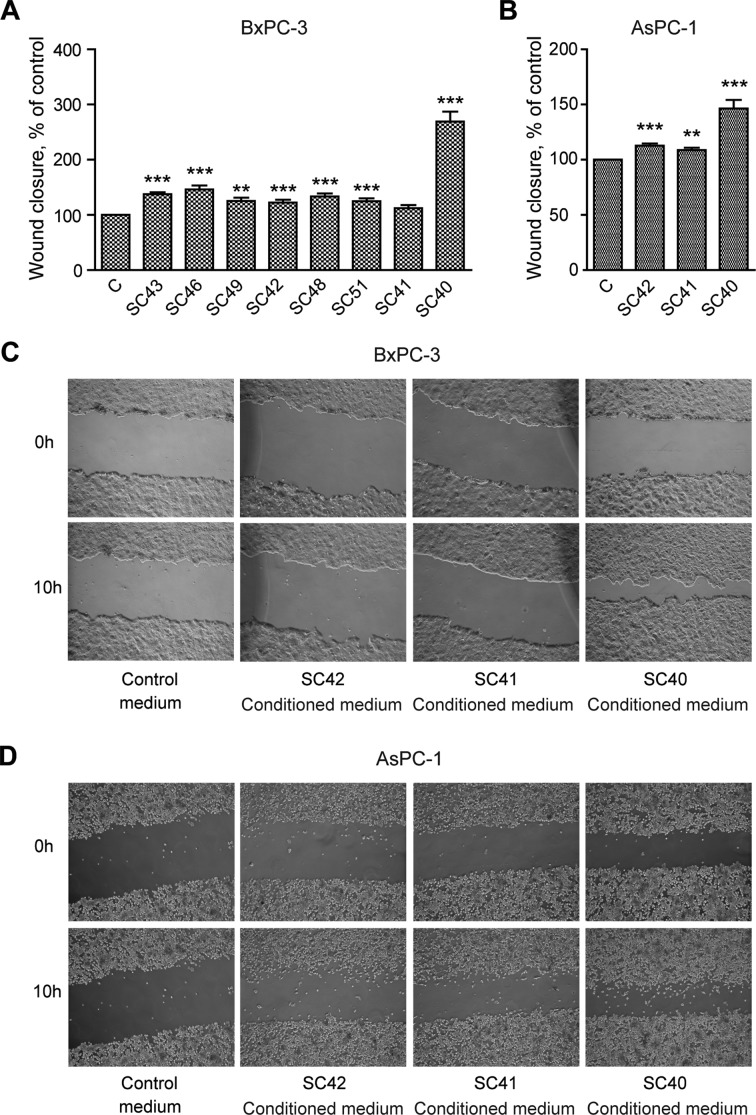
Conditioned medium from pancreatic stellate cells stimulate cancer cell migration BxPC-3 and AsPC-1 cells were cultured in colonies to confluence and scratch wounds were established in the centre of the colony. Conditioned medium from PSCs established from different PDAC patients were transferred to the BxPC-3 (**A**) and AsPC-1 (**B**) cells. The wound area was measured at 0 and 10 h (**C**–**D**) and normalized to controls. Error bars represent S.E.M.; *p < 0.05, **p < 0.005, ***p < 0.001.

We initially examined the ability of conditioned medium (CM) from eight different patient-derived PSC cell cultures to stimulate DNA synthesis and migration in BxPC-3 pancreatic cancer cells. Figure 2A shows that DNA synthesis was significantly increased above the control by conditioned medium from all of the PSC cell cultures, with a four to six-fold increase by medium from cells from four of the tumors (SC40, SC41, SC43 and SC46 cells). Essentially similar responses as in BxPC-3 cells were also found in AsPC-1 cells, when applying CM from SC40, SC41 and SC42 stellate cells (Figure 2B). Migration of BxPC-3 (Figure 3A) and AsPC-1 (Figure 3B) cells was also induced by conditioned medium from all PSC populations. However, the magnitude of the migration stimulated by the media, across the whole panel of PSCs, differed from the pattern of DNA synthesis-inducing effects. Whereas only minor effects were seen by medium from most of the PSCs, conditioned medium from SC40 cells (CM-SC40) greatly stimulated migration of BxPC-3 (Figure 3C) cells. The same general pattern was found in AsPC-1 cells (Figure 3D). These results demonstrate that conditioned media from various PSC cell preparations have the ability to induce DNA synthesis and/or cell migration, and that the degree to which these processes are enhanced depends on the specific stellate cell phenotype. To characterize these properties of the stellate cells in more detail, the SC40, SC41, and BxPC-3 cells were selected for further studies.

### Conditioned medium from PSCs phosphorylates Met in pancreatic cancer cells

**Figure 4:**
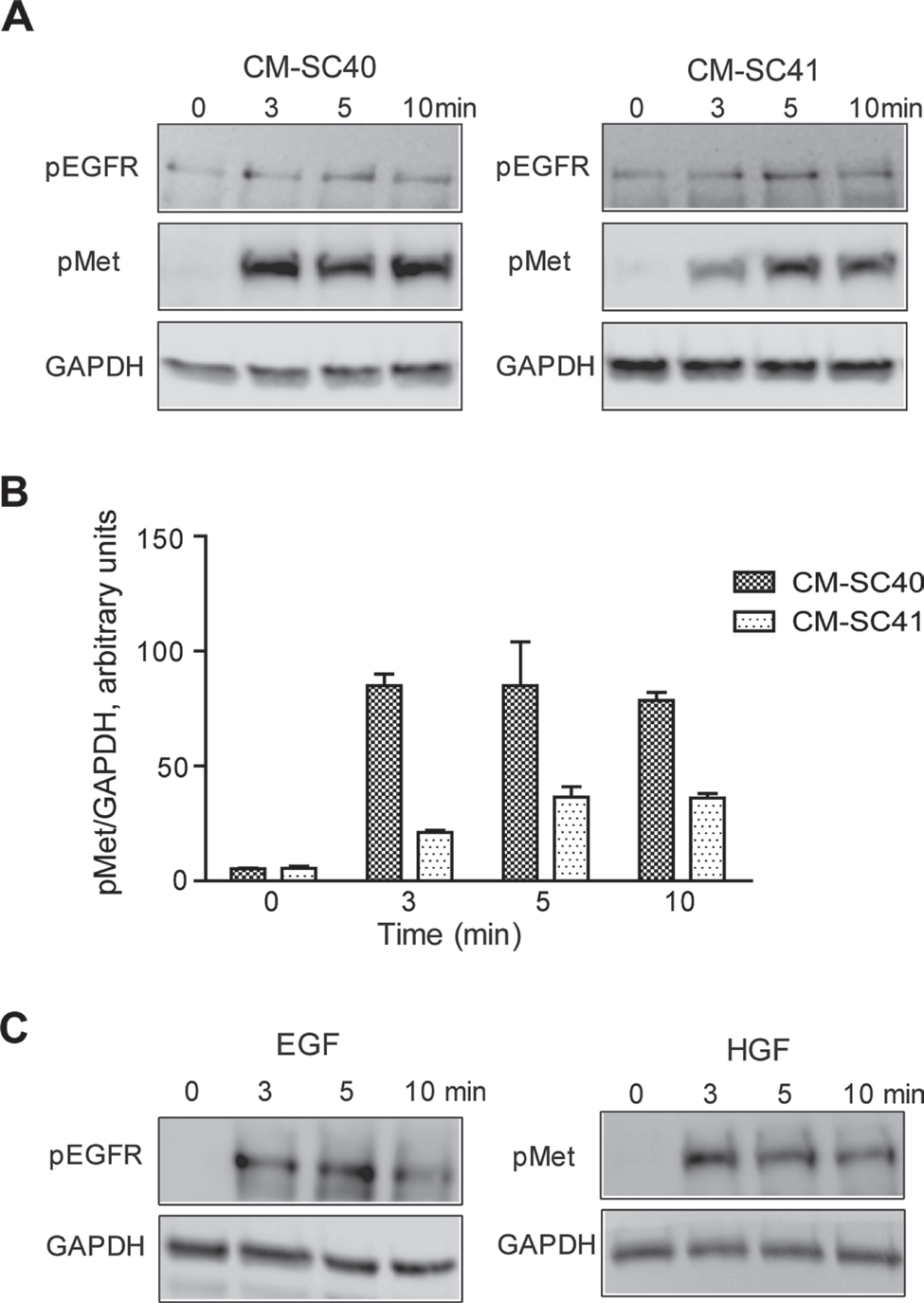
Conditioned medium from pancreatic stellate cells stimulates Met phosphorylation in pancreatic cancer cells (**A**) Conditioned medium from PSC populations SC40 and SC41 were transferred to BxPC-3 cells and incubated for 0, 3, 5 and 10 minutes. Effect of the PSCs on phosphorylation of EGFR and Met was measured by western blot and results from *one representative* experiment are shown. (**B**) The band intensity of the blots were quantified and normalized to GAPDH expression. Histograms represent mean +/−SEM of four experiments. (**C**) Phosphorylation of EGFR and Met was analysed by western blot after stimulating BxPC-3 cells for 0, 3, 5 and 10 minutes with EGF (10 nM) and HGF (1 nM). Results from *one representative* experiment are shown.

It has recently been reported that PSC-conditioned medium can activate Met in pancreatic cancer cells, although a very weak phosphorylation of Met was found [16]. We examined the phosphorylation of Met in BxPC-3 cells, using conditioned medium from two different PSCs, SC40 and SC41. Figure 4A shows that Met was phosphorylated by both CM-SC40 and CM-SC41, with the strongest signal induced by CM-SC40 (Figure 4B). These results suggest that the two conditioned media contain HGF. In contrast, little or no phosphorylation of EGFR was found (Figure 3A), suggesting that EGFR ligands were not secreted in significant amounts by these two PSCs. As controls, we also showed that EGF (10 nM) and HGF (1 nM) phosphorylated EGFR and Met, respectively (Figure 4C).

### PSCs secrete HGF into the medium, which dose-dependently activates DNA synthesis and migration

**Figure 5:**
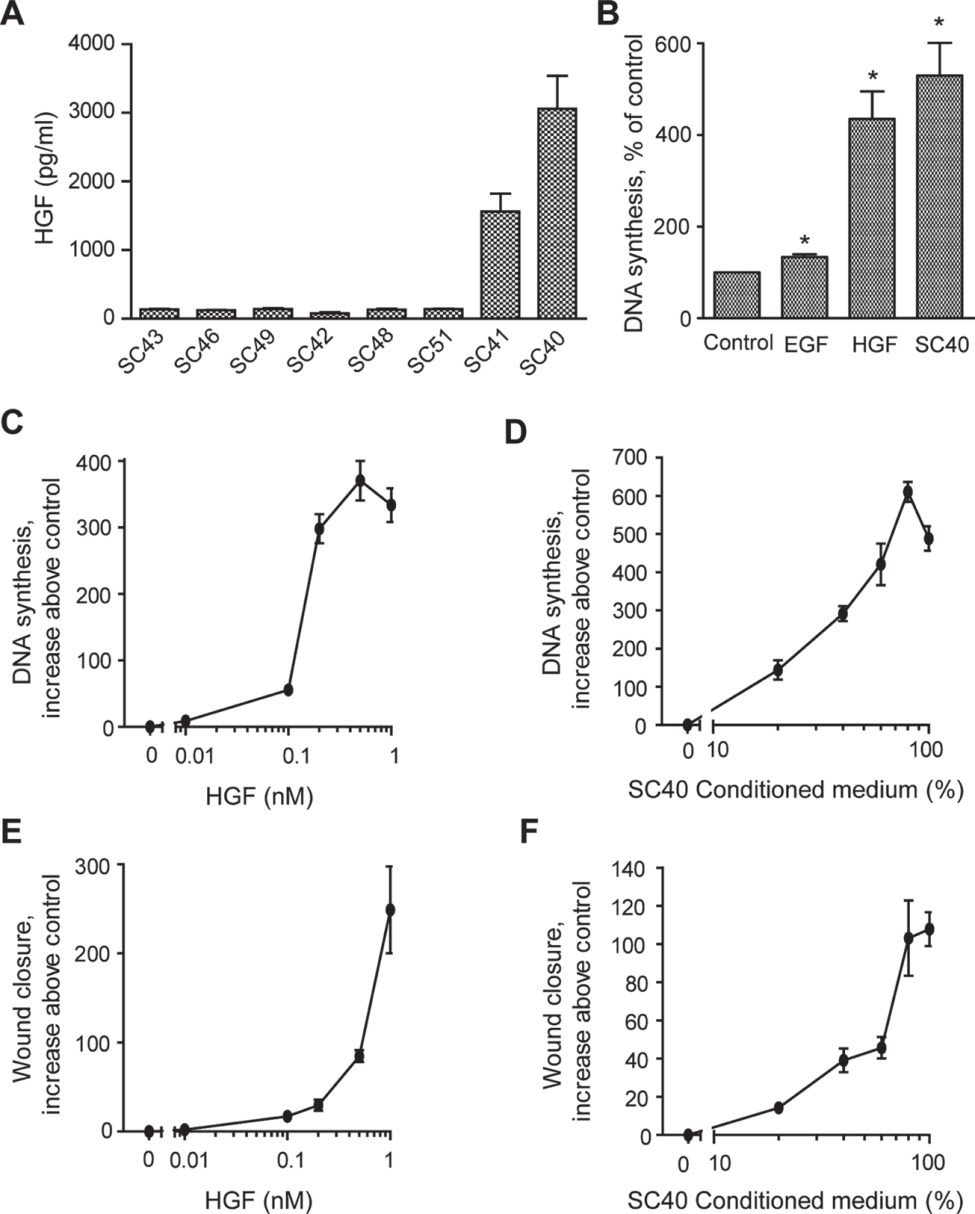
Dose dependent effects of PSC-secreated HGF on cancer cell DNA synthesis and migration (**A**) HGF secretion was measured by ELISA in conditioned medium from pancreatic stellate cell populations established from eight different PDAC patients. The results are presented in pg/ml/10^5^ cells. (**B**) The effects of EGF (10 nM), HGF (1 nM) and conditioned medium from SC40 PSCs on cancer cell proliferation was measured by DNA synthesis. Dose-dependent effects of (**C**) HGF (0–1 nM) and (**D**) SC40 conditioned medium (0–100%) on BxPC-3 DNA synthesis were analysed by measured [^3^H]-thymidine incorporation after 24 h of incubation. Dose-dependent effects of (**E**) HGF (0–1 nM) and (**F**) SC40 conditioned medium (0–100%) on BxPC-3 migration were analysed by wound assay after 10 h of incubation. The wound area was measured at 0 and 10 h and normalized to controls. Error bars represent S.E.M.; *p < 0.05, **p < 0.005, ***p < 0.001.

We next studied the HGF secretion by the whole panel of the eight PSCs. The results show that the SC40 and SC41 cells expressed very high levels of HGF (approximately 3000 and 1500 pg/ml, respectively), compared to the other PSC cells (120–150 pg/ml) (Figure 5A). Conditioned medium from the high-HGF producing SC40 cells stimulated DNA synthesis to the same level as HGF (Figure 5B). We also found that EGF was a weak inducer of DNA synthesis in BxPC-3 cells, as previously reported by others [23]. Figure 5C shows the dose-dependency of the effect of HGF on DNA synthesis in the BxPC-3 cells. Increasing concentrations of CM-SC40, which expressed the highest level of HGF among the different media, showed similar dose-dependent effects as HGF on BxPC-3 cell DNA synthesis (Figure 5D). Moreover, the impact of different concentrations of HGF on BxPC-3 migration was studied in a wound closure model. The migration of BxPC-3 cells was dose-dependently enhanced by HGF and increasing concentrations of CM-SC40 showed comparable dose-dependent effects (Figure 5E and 5F). It may be noted that, as compared to the effects on DNA synthesis, simulation of migration consistently required higher concentrations of CM-SC40 (as well as of HGF).

### Conditioned medium-stimulated DNA synthesis and migration of pancreatic cancer cells is inhibited by blocking Met

**Figure 6:**
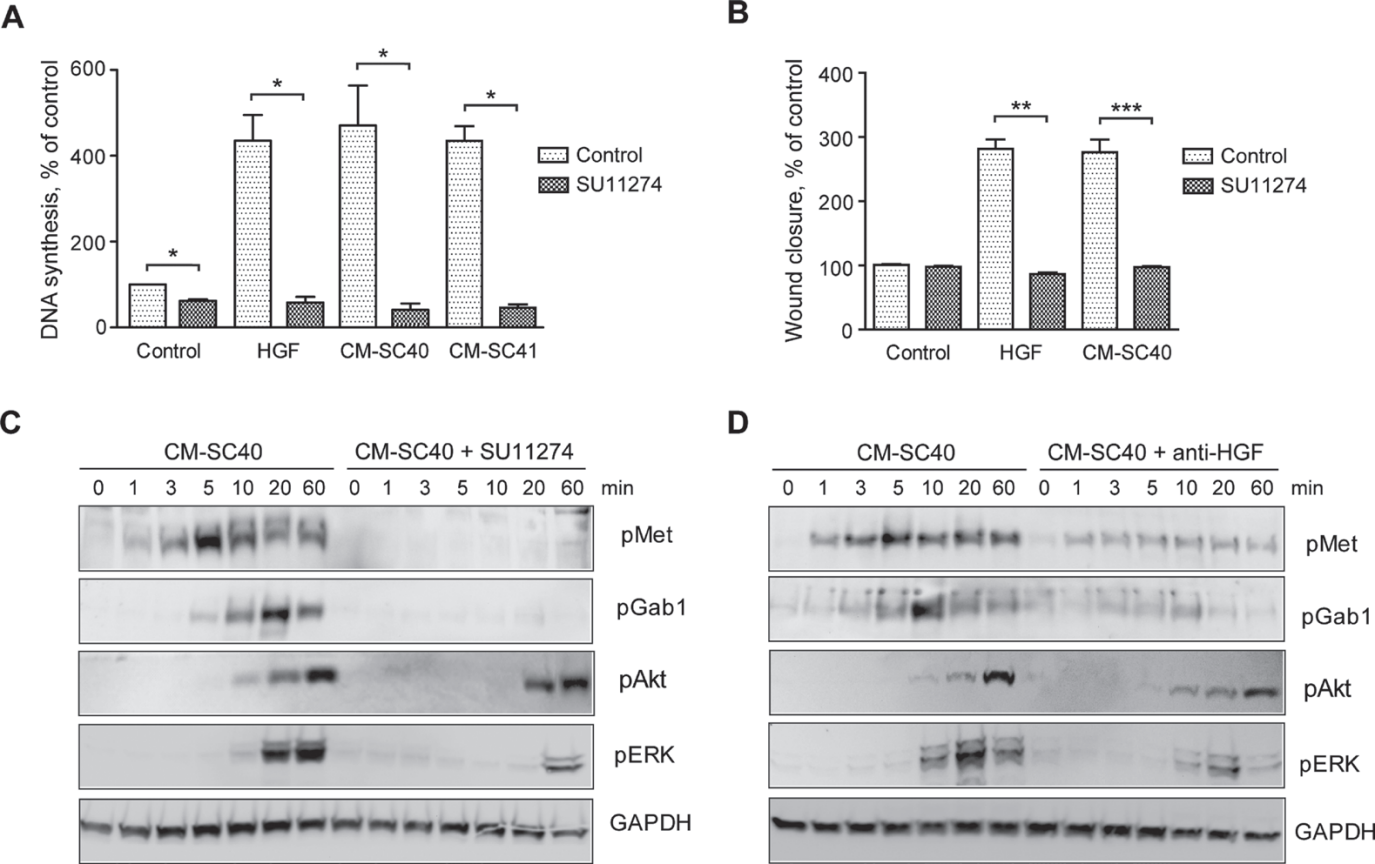
Pancreatic stellate cell-induced cancer cell migration and DNA synthesis are inhibited by blocking HGF signaling (**A**) DNA synthesis was determined by measuring [^3^H]-thymidine incorporation in BxPC-3 cells stimulated with HGF or conditioned medium from the SC40 and SC41 PSCs with or without the Met signaling inhibitor SU11274 (2 μM). HGF and SU11274 were added at 48 h, [^3^H]-thymidine after 68 h and the cells were harvested at 72 h. SC40 and SC41 data were conducted from independent experiments (**B**) Cancer cell migration was determined by wound assay after 10 h stimulation of BxPC-3 cells with HGF or conditioned medium from the SC40 PSCs with or without the Met signaling inhibitor SU11274. (**C**–**D**) BxPC-3 cells were stimulated with conditioned medium from the PSC cells SC40 for the indicated time points 30 min after adding (C) SU11274 (2 μM) or (D) HGF neutralizing antibody (10 μg/ml). The cells were lysed and proteins subjected to immunoblotting using the indicated antibodies. Immunoblots shown are representative of three independent experiments. Error bars represent S.E.M.; *p < 0.05, **p < 0.005, ***p < 0.001.

Since the PSCs express other factors than HGF that might stimulate DNA synthesis and migration of BxPC-3 cells, we blocked Met activity using the Met-specific tyrosine kinase inhibitor SU11274. Both DNA synthesis and cell migration of BxPC-3 cells induced by CM-SC40 were inhibited down to the level of the control (Figure 6A and 6B). DNA synthesis induced by CM-SC41 was inhibited by SU11274 in a similar manner. These results strongly suggest that DNA synthesis and cell migration were mediated through the activation of Met. By immunoblotting, we showed that SU11274 inhibited phosphorylation of Met by CM-SC40 (Figure 6C). Furthermore, SU11274 abolished the phosphorylation of the docking protein Gab1, which has a particular role in MET signaling [24, 25], and greatly reduced the ERK phosphorylation. Additional evidence that HGF was the active factor in CM-SC40 was obtained in experiments where the medium was pre-incubated with an antibody against HGF, before added to the BxPC-3 cells. As shown in Figure 6D, this reduced the phosphorylation of Met and of Gab1 and ERK. These results provide further support for the conclusion that DNA synthesis and cell migration are induced through Met activated by HGF secreted by the SC40 stellate cells.

### IL-1α stimulation of PSCs increases the level of HGF secretion and conditioned medium-stimulated DNA synthesis and migration of pancreatic cancer cells

**Figure 7:**
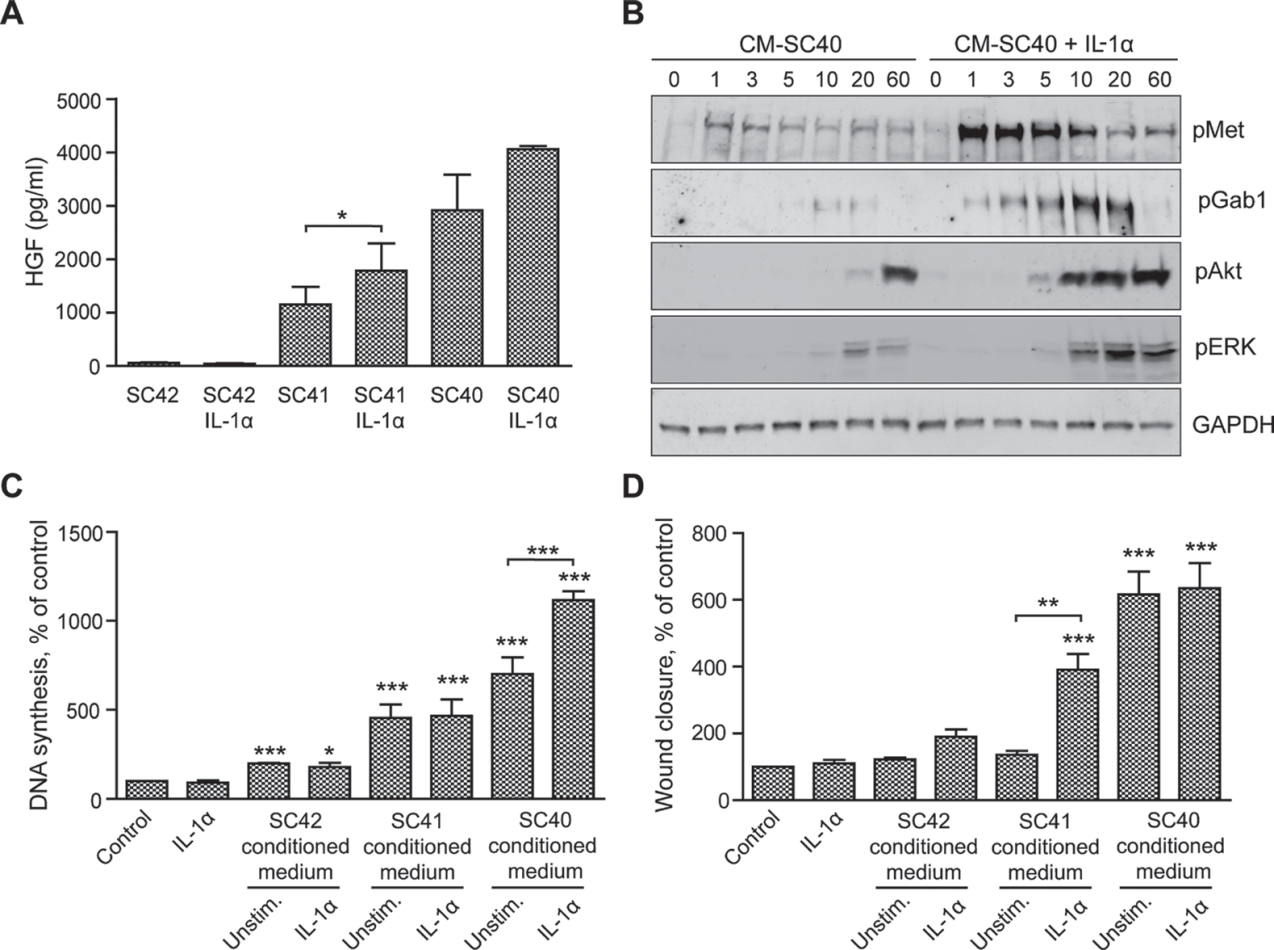
IL-1α-stimulated PSCs increase HGF secretion and enhance cancer cell migration and DNA synthesis (**A**) HGF secretion was measured by ELISA in conditioned medium from pancreatic stellate cells SC40, SC41 and SC42, incubated in the presence or absence of IL-1α (1 ng/ml). (**B**) BxPC-3 cells were stimulated for the indicated time points with conditioned medium from the PSCs (SC40) cultured in the presence or absence of IL-1α (1 ng/ml). The cells were lysed and proteins subjected to immunoblotting using the indicated antibodies. Immunoblots shown are representative of three independent experiments. (**C**) The effect of IL-1α stimulated pancreatic stellate cells on cancer cell DNA synthesis was analysed by measuring [^3^H]-thymidine incorporation. BxPC-3 cells were stimulated for 24 hours with IL-1α (1 ng/ml) or with conditioned medium obtained from PSCs cultured in the presence or absence of IL-1α (1 ng/ml), with [^3^H]-thymidine added at 18 hours. The results are presented as mean +/−SEM normalized to unstimulated control. (**D**) BxPC-3 cells were cultured in colonies to confluence and scratch wounds were established in the centre of the colony. Control medium including IL-1α (1 ng/ml) or conditioned medium from PSC cultured in the presence or absence of IL-1α (1 ng/ml) was transferred to the wound assays. The wound area was measured at 0 and 10 h and normalized to controls. Direct effects of IL-1α were measured in independent experiments. Error bars represent S.E.M.; *p < 0.05, **p < 0.005, ***p < 0.001.

We previously demonstrated that IL-1α increases the ability of pancreatic stellate cells to enhance migration of pancreatic cancer cells [20]. In the present work we aimed to further investigate this effect of IL-1α. Figure 7A shows that IL-1α increased HGF secretion both in the high HGF-producing cells (SC40) and intermediate HGF-producing cells (SC41), but not in the low-producing cells (SC42). Phosphorylation of Met and the downstream signaling molecules Gab1, Akt and ERK was increased when BxPC-3 cells were stimulated by conditioned medium from IL-1α-stimulated SC40 PSCs (Figure 7B), suggesting a functional HGF response to IL-1α. Furthermore, IL-1α-stimulated SC40-conditioned medium enhanced DNA synthesis in BxPC-3 cells whereas no such effect was detected by stimulation with conditioned medium from SC42 or SC41 cells (Figure 7C). IL-1α alone did not affect DNA synthesis or cell migration of BxPC-3 cells (Figure 7C and 7D). However, IL-1α enhanced SC41-stimulated migration in the BxPC-3 cells. No further stimulation could be elicited by IL-1α treatment of the SC40 cells (Figure 7D), suggesting that maximal HGF-induced migration was reached in these cells even in the absence of IL-1α.

### TGFβ stimulation of PSCs reduces the level of HGF secretion and conditioned medium-stimulated DNA synthesis and migration of pancreatic cancer cells

**Figure 8:**
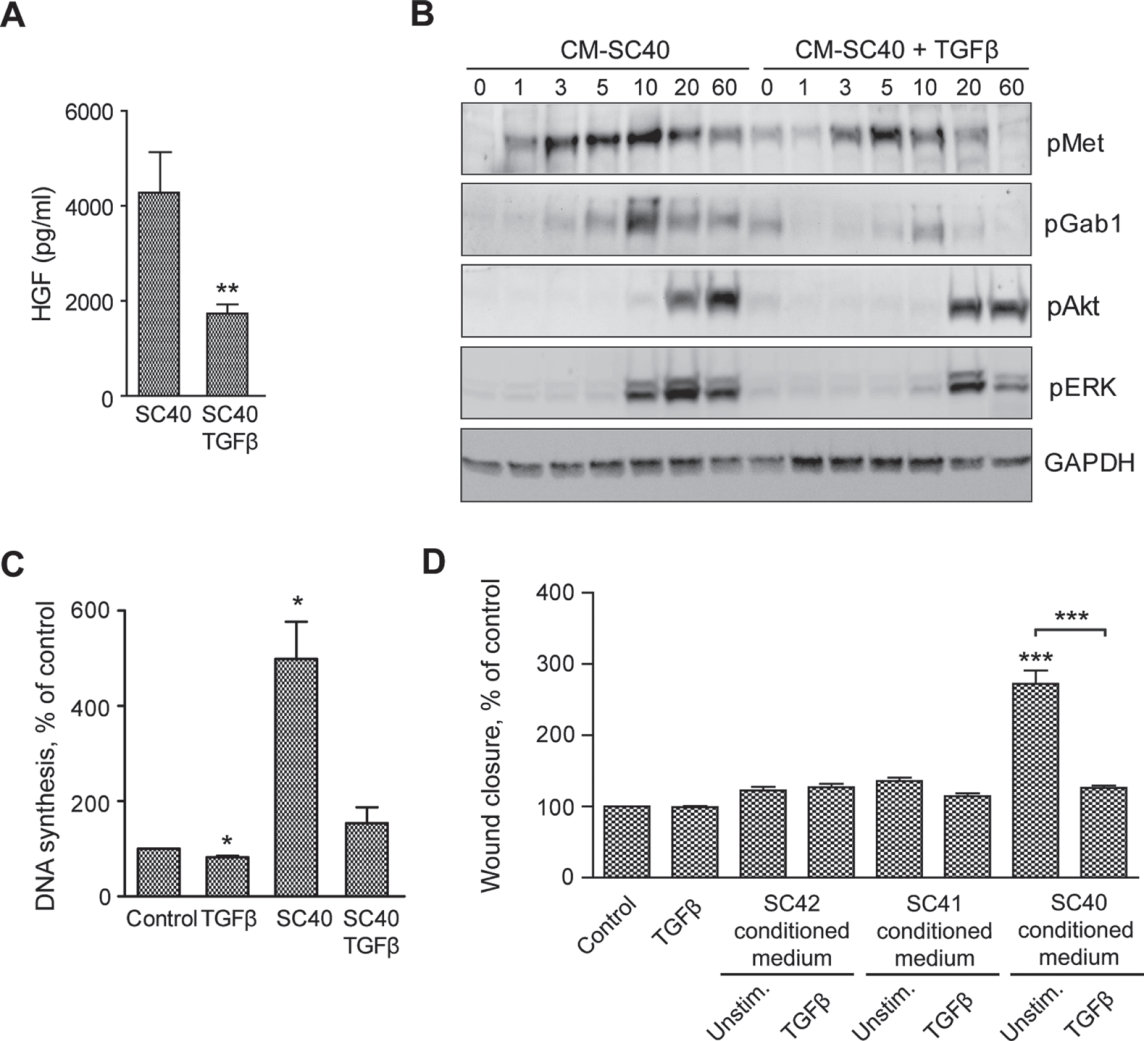
TGFβ-stimulated PSCs reduces HGF secretion and enhance cancer cell migration and DNA synthesis (**A**) HGF secretion was measured by ELISA in conditioned medium from the pancreatic stellate cells SC40, incubated in the presence or absence of TGFβ (2 ng/ml). The results are normalized to unstimulated controls. (**B**) BxPC-3 cells were stimulated for the indicated time points with conditioned medium from the PSCs (SC40) cultured in the presence or absence of TGFβ (2 ng/ml). The cells were lysed and proteins subjected to immunoblotting using the indicated antibodies. Immunoblots shown are representative of three independent experiments. (**C**) DNA synthesis was determined by measuring [^3^H]-thymidine incorporation in BxPC-3 cells stimulated with TGFβ (2 ng/ml) or conditioned medium from the pancreatic stellate cells SC40, incubated in the presence or absence of TGFβ (2 ng/ml). TGFβ and PSC conditioned medium were added at 48 h, [^3^H]-thymidine after 68 h and the cells were harvested at 72 h. (**D**) Cancer cell migration was determined by wound assay after 10 h stimulation of BxPC-3 cells with TGFβ or conditioned medium from the pancreatic stellate cells SC42, SC41 and SC40, incubated in the presence or absence of TGFβ (2 ng/ml). The wound area was measured at 0 and 10 h and normalized to controls. Error bars represent S.E.M.; *p < 0.05, **p < 0.005, ***p < 0.001.

We have reported inhibitory effects of TGFβ on PCS-mediated stimulation of pancreatic cancer cell migration [20]. Here we demonstrate that TGFβ stimulation of PSCs reduced HGF secretion (Figure 8A). Furthermore, as demonstrated by immunoblotting, phosphorylation of Met and the downstream signaling molecules Gab1 and ERK was reduced when BxPC-3 cells were stimulated by conditioned medium from TGFβ-stimulated PSCs (Figure 8B), suggesting that this is caused by a reduction in the level of HGF in the conditioned medium. TGFβ did not affect DNA synthesis or cell migration of BxPC-3 cells (Figure 8C and 8D). In contrast, DNA synthesis and migration stimulated by conditioned medium from TGFβ-treated SC40 PSCs was greatly reduced compared to medium from unstimulated SC40 PSCs (Figure 8C and 8D). Cancer cell migration stimulated by conditioned medium from intermediate (SC41) and low (SC42) HGF-producing PSCs was not inhibited by TGFβ treatment of the PSCs (Figure 8D). These results suggest that TGFβ, by decreasing HGF secretion from the PSCs, can reduce DNA synthesis and migration of pancreatic cancer cells.

## DISCUSSION

Investigations of the contribution of stromal fibroblastic cells to the pathobiology of pancreatic cancer have mostly utilized various forms of immortalized fibroblast cell lines [26]. However, some observations have indicated that immortalized and primary PSCs behave differently in terms of functional responses [27, 28]. Furthermore, presumed heterogeneity in PSCs from different patients has been suggested to be associated with the heterogeneous nature of pancreatic cancer [5]. In the present study, we have isolated primary pancreatic stellate cells from eight different human pancreatic adenocarcinomas and studied their effects on pancreatic cancer cells, particularly the role of PSC-derived HGF in stimulation of proliferation and migration. We show that (i) the different PSC populations were heterogeneous, as they exhibited a wide range of variation with respect to their ability to stimulate the carcinoma cells and to secrete HGF, (ii) PSC-induced stimulation of cancer cells can be mediated by the HGF/Met signaling pathway, and (iii) in HGF-secreting PSCs, IL-1α and TGFβ can regulate the HGF levels as well as the HGF-mediated effects on the cancer cells.

HGF has been implicated in the complex interplay between malignant epithelial cells and non-malignant mesenchymal-derived cells residing in the fibro-inflammatory tumor stroma of pancreatic cancers [16]. Previously, some studies have reported stimulatory effects on pancreatic cancer cell migration by HGF secreted from immortalized fibroblast cells lines [17, 29] or primary fibroblasts from pancreatic tumors [13]. To our knowledge, specific demonstration and comparison of HGF production from separate primary pancreatic stellate cell preparations isolated from different patients and their effects on pancreatic cancer cells in terms of DNA synthesis and migration has not previously been reported. We found that PSCs from two particular tumors (SC40 and SC41) secreted very high levels of HGF (3,000 and 1,500 pg/ml, respectively), whereas the remaining six PSC populations secreted broadly similar low levels of HGF (120–150 pg/ml). The highest HGF-secreting PSC population, SC40, was also most potent in inducing cancer cell migration and DNA-synthesis. Furthermore, the SC41 cells revealed interesting additional details about the regulation and functional role of HGF in the cellular interactions studied here. Thus, while we have previously shown that IL-1α is abundantly present in the malignant cells in PDAC and regulates PSC production of various ECM components [30], and enhances the ability of PSCs to stimulate pancreatic cancer cells [20], the present results indicated that IL-1α also increased the HGF production in SC41 cells up to a level that permitted stimulation of BxPC-3 cell migration. This did not occur in the SC42 cells, adding to the evidence of heterogeneity.

The fact that the effect of conditioned medium (CM-SC40) on DNA synthesis and migration in BxPC-3 cells was blocked by the Met-specific tyrosine kinase inhibitor (SU11274) lends further support to the conclusion that HGF is the major active substance released from the PSCs. Furthermore, both SU11274 and the neutralizing HGF antibody (MAB294) inhibited CM-SC40-induced Met phosphorylation. The inhibition by MAB294 was not as strong as for SU11274, which might be due to incomplete blockade by MAB294 of the HGF-Met interaction, as has recently been reported for another HGF antibody [31]. Gab1 is a binding partner of Met and is of particular importance in the downstream signaling pathways, mediating mitogenic signals from this receptor [24, 25]. Both SU11274 and MAB294 significantly reduced phosphorylation of Gab1 and ERK. This suggests that Gab1 is phosphorylated by HGF present in the conditioned medium and that ERK activation is dependent on Gab1. In contrast, Akt phosphorylation was only slightly affected, suggesting that other components than HGF in the medium may account for the Akt activation. In addition to the direct activation of Met by HGF, Met may be activated through transactivation by other receptor tyrosine kinases, such as EGFR, or by G-protein-coupled receptors [32]. Since EGFR was not phosphorylated by CM-SC40 or CM-SC41, transactivation by EGFR seems unlikely to account for the activation of Met in these cells. Finally, the direct demonstration of HGF concentrations in the conditioned mediums substantiates HGF as the active agent.

TGFβ inhibits IL-1α-mediated PSC stimulation of pancreatic cancer cell migration [20]. Furthermore, in a study utilizing immortalized fibroblasts, it has been suggested that TGFβ negatively regulates HGF expression and HGF-induced cancer cell invasion [29]. In the present study, TGFβ reduced HGF production in PSCs and reduced cancer cell mitogenesis and migration induced by the high HGF-producing PSCs concomitant with a reduction in phosphorylation of Gab1 and ERK in the cancer cells. This indicates that the previously reported inhibitory effect of TGFβ on PSC-induced cancer cell migration involves HGF-dependent signaling. It is of great interest to note that the present results, showing that TGFβ had no direct effect on the carcinoma cells but inhibited their activity via the stellate cells, are consistent with the fact that BxPC-3 cells have mutations in the TGFβ pathway [33], abolishing the tumor suppressor effect of TGFβ, while these mutations are not likely to exist in the stellate cells. Recent genomic analyses of human pancreatic cancers have revealed a mutation frequency of up to approximately 50% in the TGFβ signaling pathway [34, 35]. Heterogeneity in TGFβ pathway mutations has also recently been shown to influence various characteristics of PDAC stroma such as tissue tension and tumor stroma interactions [36].

There is a controversy in the literature regarding the importance of the pancreatic stroma for the progression of pancreatic cancer as well as its role as a target for therapy [9, 10, 37, 38]. Recently inhibition of the HGF/Met pathway mediating tumor-stroma interactions was reported to inhibit local tumor growth in an orthotropic pancreatic cancer model [16]. Our data on HGF secretion from pancreatic stellate cells suggest that the complexity of signal communication between stromal cells and cancer cells is further complicated by the existence of differentially expressed signaling pathways among stellate cells isolated from different patients. These observations might be of importance for the design of future experimental and potential clinical trials or treatment regimens targeting tumor-stroma interactions in pancreatic cancer. The present demonstration of a functional heterogeneity of PSCs in terms of HGF-mediated tumor-stroma interactions suggests that inhibition of the HGF pathway as a novel treatment approach in PDAC might have different effects in different subsets of patients.

## MATERIALS AND METHODS

### Patients

The study protocol and patient consent documents were approved by the Regional Committee for Medical and Health Research Ethics (REC South East, project number 2010/694a), and was in compliance with the Helsinki Declaration. Written informed consent was obtained from all study participants. The study included only adults.

### Materials

HGF (recombinant human) was obtained from Millipore (Billerica, MA), EGF (recombinant human) from Sigma Chemical Co. (St. Louis, MO), IL-1α (recombinant human) from Biolegend (San Diego, CA), TGFβ (recombinant human) from R&D Systems Europe (Abingdon, UK), Human anti-HGF (MAB294) from R&D Systems Europe (Abingdon, UK), anti-alpha smooth muscle actin (αSMA) (BS66) from Nordic Biosite AB (Taby, Sweden) and SU11274 from Sigma-Aldrich (Oslo, Norway). Anti-phospho-EGFR (Tyr1173) was from Life Technologies/Invitrogen (Carlsbad, CA). Anti-phospho-Met (Tyr1234/1235), anti-phospho-Gab1 (Tyr627), anti-phospho-Akt (Ser473), anti-phospho-ERK1/2 (Thr202/Thr204), anti-vimentin (D21H3), anti-glial fibrillary acidic protein (GFAP) (GA5), anti-vinculin and anti-GAPDH were from Cell Signaling (Beverly, MA). Secondary goat anti-mouse and goat anti-rabbit IgG HRP-conjugated antibodies were purchased form Bio-Rad Laboratories (Hercules, CA). [^3^H]-thymidine (20–30 Ci/mmol) was from Perkin Elmer (Waltham, MA). All other chemicals used were of analytical quality.

### Cells, isolation and culture

Human pancreatic stellate cells (PSCs) were isolated from pancreatic tumor tissue obtained during pancreatic surgery from patients with resectable pancreatic head adenocarcinoma and cultured by the outgrowth method developed by Bachem et al. [39] as explained elsewhere [27]. Cultures from tumors from eight different patients were established and propagated, designated as SC40, SC41, SC42, SC43, SC46, SC48, SC49 and SC51 cells. The purity of the PSCs from each tumor was assessed by morphology and demonstration of αSMA and Vimentin expression. All experiments were performed using cell populations between passage 4 and 8. BxPC-3 and AsPC-1 were purchased from ATCC (Manassas, VA, USA). All cells were cultured in 6 wells plates in Dulbecco's modified Eagle's medium containing 4.5 g/l glucose (DMEM). The media were supplemented with 100 μg/ml Pen-Strep, Glutamax and 10 % fetal bovine serum (FBS) (Life Technologies). Serum free conditioned medium from PSCs cultured to confluency with or without IL-1α (1 ng/ml) or TGFβ (2 ng/ml) were harvested after 5 days of culture and stored at −20°C until use.

### Immunocytochemistry

Cultured PSCs were fixed in 3% formalin and immunostained over night with anti-alpha smooth muscle actin (BS66) and anti-vimentin (D21H3). Positive cells were visualized by Alexa Fluor 488 and Alexa Fluor 594 conjugated secondary antibodies from Jackson ImmunoResearch (West Grove, PA) and DAPI from Jackson ImmunoResearch was used for nuclear staining.

### DNA synthesis

DNA synthesis induced by PSC-conditioned medium, HGF and EGF was measured by thymidine incorporation as previously described [27]. Briefly, cells were seeded in 12-well plates and grown for 24 hours in complete medium. Thereafter they were grown in medium without serum for 24 hours. The cells were then stimulated as indicated for 24 h and pulsed with [^3^H]-thymidine (1 μCi/ml) the last 4 hours of stimulation.

### Cell migration assay

Cell migration was assessed using a scratch assay [40]. 2 × 10^5^ BxPC-3 cells in 100 μl DMEM medium supplemented with 10% FBS were seeded in 12 well culture plates pre-marked with three ink marks under the bottom of each well. The cells were left to adhere for 2 h; then, 1 ml of serum free medium (SF) was added, and the cells incubated overnight to confluence. A scratch was made with a 100 μl pipette tip. The marks under the dishes served to ensure that exactly the same observation field was studied during the observation period. After scratching, the cells were washed twice with NaCl and then kept in SF medium or PSC conditioned medium for 10 h. The scratch wounds were observed in a Zeiss Axiovert 25 inverted microscope with a 5× objective (Carl Zeiss AS, Oslo, Norge). Images (each 1.4 × 1.0 mm), taken before the addition of SF or PSC supernatants and at 10 h, were obtained with a Zeiss AxioCam ICc3 (Carl Zeiss AS). For each picture, the wound area was measured by FIJI software as described by Schindelin et al [41]. Per cent wound closure was calculated for the time point of observation based on the mean of 2–3 observations from each scratch.

### Immunoblotting

Cells were stimulated with PSC-conditioned medium, EGF or HGF as indicated after 24 h of serum starvation. Culturing of PSCs on matrigel (BD Bioscience, Bedford MA) was performed in DMEM plus 5% FBS in either the presence or absence of 2.5 mM N-Acetylcysteine (NAC) (R&D Systems Europe, Abingdon, UK) for 6 days. Cells were washed with PBS and total cell lysates prepared by boiling for 5 min in Laemmli buffer (4% SDS, 20% glycerol and 120 mM Tris-HCl, pH 6.8) with the addition of 0.02% bromophenol blue (BFB) and 5% β-mercaptoethanol. Aliquots of 15μg protein were separated on 10% polyacrylamide gels by electrophoresis (SDS-PAGE). The proteins were transferred to nitro-cellulose membranes using a semidry transfer system (Bio-Rad). The membranes were blocked in Tris-buffered saline containing 0.1% Tween 20 (TBST) with 5% non-fat dry milk solution and incubated with the primary antibodies as indicated (in TBST with 5% non-fat dry milk or BSA) overnight at 4°C. The blots were then washed 3 times in TBST and incubated with HRP-conjugated secondary antibodies at room temperature for 1 h. The blots were visualized with LumiGLO^®^ (KPL, Gaithersburg, MD). Densitrometic analyses of immunoblots were obtained with Labworks Software (UVP, Cambridge, UK).

### ELISA

The levels of HGF in conditioned medium from pancreatic stellate cells were assessed after culturing the cells for 3 days in serum-free DMEM medium. The conditioned medium was harvested and the concentration of HGF (Nordic BioSite, Oslo, Norway) was measured by ELISA according to the manufacturers’ protocol.

### Statistical analysis

The statistical analysis was performed with GraphPad Prism 5 (GraphPad Software), *p* < 0.05 was considered statistically significant and error bars throughout indicate standard error of the mean (SEM). Normalized data were analysed by paired *t*-test, multiple comparisons were analysed using ANOVA including Bonferroni correction.
